# Diet of breeding Eleonora's falcon *Falco eleonorae* in Algeria: Insights for the autumn trans‐Mediterranean avian migration

**DOI:** 10.1002/ece3.9065

**Published:** 2022-07-05

**Authors:** Boudjéma Samraoui, Yves Kayser, Laïd Touati, Farrah Samraoui, Abdennour Boucheker, Hamed A. El‐Serehy, Kenz Raouf Samraoui

**Affiliations:** ^1^ Laboratoire de Conservation des Zones Humides University 8 mai 1945 Guelma Algeria; ^2^ Biology Department University Badji Mokhtar Annaba Algeria; ^3^ Research Institute for the Conservation of Mediterranean Wetlands Tour du Valat Le Sambuc France; ^4^ Biology and Ecology Department University of Constantine Constantine Algeria; ^5^ Department of Zoology, College of Science King Saud University Riyadh Saudi Arabia; ^6^ Faculty of Science Jihočeská Univerzita České Budějovice Czech Republic; ^7^ Department of Functional Ecology, Institute of Botany The Czech Academy of Sciences Třeboň Czech Republic

**Keywords:** autumn migration, diet, passerine, trans‐Mediterranean migration, trans‐Saharan migrants

## Abstract

How environmental changes are affecting bird population dynamics is one of the most challenging conservation issues. Dietary studies of top avian predators could offer scope to monitor anthropogenic drivers of ecosystem changes. We investigated the diet of breeding Eleonora's falcon in an area of Northeastern Algeria in the years 2010–2012. Feathers and insect remains originating from prey plucking behavior were analyzed, providing insights into the seasonally changing diet of this raptor, as well as the trans‐Mediterranean avian migration. A total of 77 species of birds (16 Sylviidae, 11 Turdidae, and 4 Emberizidae), 3 species of insects, and 1 lizard were identified among prey remains, reflecting a diverse diet. Diet composition and prey abundance varied seasonally, faithfully correlating with the passage of migrant birds as recorded from bird ring recoveries. Our findings suggest that dietary studies of predators might be deployed to investigate changes in bird migration. We discuss our results in the context of trans‐Mediterranean migration, with early‐season prey mainly comprising trans‐Saharan migrants (*Apus apus* and *Merops apiaster*) and late‐season prey being dominated by Mediterranean winter migrants (*Erithacus rubecula*, *Turdus philomelos*, *Sylvia atricapilla*, and *Sturnus vulgaris*). Notably, we observed a significant reduction in species richness of passerine remains in 2012, potentially highlighting a decline in the diversity of avian migrants.

## Graphical abstract

We investigated the diet of breeding Eleonora’s Falcon in an area of North‐eastern Algeria in the years 2010‐2012. Diet composition and prey abundance varied seasonally, faithfully correlating with the passage of migrant birds. We discuss our results in the context of trans‐Mediterranean migration, with early‐season prey mainly comprising trans‐Saharan migrants and late‐season prey being dominated by Mediterranean winter migrants.

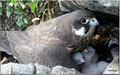

## 1 INTRODUCTION

During their remarkable journey, migrant organisms move regularly through the “energy landscape” and the “landscape of fear” (Gallagher et al., 2017; Laundré et al., 2001). For migrant prey, the spatio‐temporal heterogeneity of resource variability and predation risks implies trade‐offs between energetics requirements (Shepard et al., 2013) and vulnerability to predation (Martin et al., 2015). Thus, natural selection, through the ecological process of migration, shapes the eco‐evolution of predators and their prey (Burak et al., 2018; Johnson & Belk, 2020). Understanding how anthropogenically driven changes are potentially affecting predator–prey population dynamics is crucial to prioritize conservation actions (Buechley et al., 2019; Runge et al., 2015).

Eleonora's falcon (*Falco eleonorae*) is a colony‐living raptor that winters on the east coast of Africa, but returns to its Mediterranean breeding grounds in April/May (Ristow & Wink, 1995). The species also breeds in the adjacent Atlantic Ocean, along the North African coast (Walter, 1979). It is a late‐season breeder, with the nestling period timed to coincide with postnuptial avian migration. The species is fairly abundant in the eastern Mediterranean, but it breeds sparingly on a few rocky islets spread along the entire North African coast. Its biology has been the focus of several studies (reviewed in Walter, 1979), but knowledge of the status and ecology of Algerian populations is limited (but see Bakour & Moulaï, 2019; Peyre et al., 2018; Samraoui et al., 2011; Touati et al., 2017).

**FIGURE 1:**
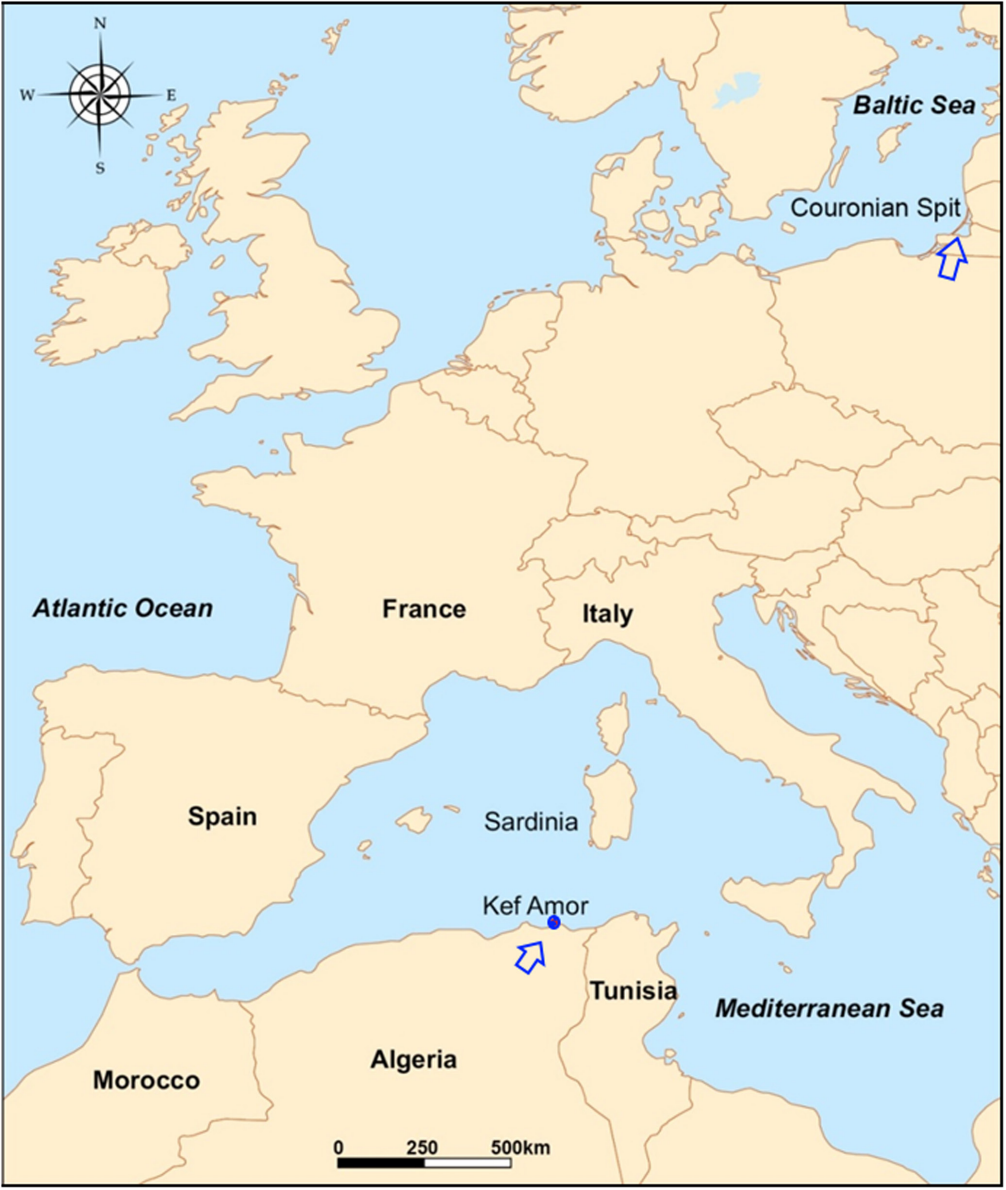
The Western Mediterranean Basin showing the location of the islet of Kef Amor, Northeastern Algeria

The diet of Eleonora's falcon has been investigated across its range (Ristow et al., 1986; Spina et al., 1987; Walter, 1979), revealing considerable differences in prey composition that potentially may be linked to variation in the migratory patterns of prey. In the context of environmental changes that are inducing a rapid decline in migratory songbirds (Both et al., 2009; Rosenberg et al., 2019) and given that the ecology of Eleonora's falcon is likely intimately linked to prey migration, we aimed to provide insights into the relationships between prey remains and the trans‐Mediterranean migration of songbirds (Passeriformes) and non‐passerines in Northeastern Algeria (Figure 1) by (1) analyzing the diet of Eleonora's falcon during the nestling phase, and (2) testing whether diet of Eleonora's falcon reflects the known passage of migrant birds. The information derived from this study can be used to develop a long‐term monitoring scheme of the avian trans‐Mediterranean migration and inform conservation efforts.

## 2 MATERIALS AND METHODS

### 2.1 Study area

This study was carried out at Kef Amor (37°5.07’N, 7°19.87′E), a rocky islet close to Chetaïbi in Northeastern Algeria (Samraoui & Samraoui, 2008; Touati et al., 2017) (Figure 1, Table 1). Besides the breeding colony of Eleonora's falcon, the islet hosts breeding populations of Yellow‐legged Gull *Larus michahellis* and Scopoli's Shearwater *Calonectris diomedea*. The climate of the study area is typically Mediterranean with characteristically two contrasting periods: a hot and dry season stretching from May to October and a cool and rainy season from November to April.

**TABLE 1:** Sampling dates at Kef Amor during three breeding seasons (2010–2012) with number of samples shown in parentheses

2010	2011	2012
12 September (49)	13 September (53)	20 July (1)
18 September (38)	28 September (23)	27 July (8)
30 September (29)	5 October (19)	3 August (8)
8 October (20)		11 August (15)
		17 August (21)
		26 August (27)
		30 August (20)
		6 September (23)
		11 September (25)
		18 September (24)
		26 September (24)
		3 October (23)
		10 October (23)
		17 October (20)
		24 October (20)

### 2.2 Sampling

We collected a total of 513 dietary samples, consisting of bird feathers and insect wings gathered from pluck sites near nests, across three breeding seasons (2010–2012). All remains at a pluck site gathered together represented a sample and, within each sample, prey items were identified and counted. We could unequivocally assign 503 samples to active (containing at least one egg) nests. Weekly sampling was carried out in 2012 (*N* = 282 samples), but logistical constraints only permitted limited sampling in both 2010 (*N* = 136 samples) and 2011 (*N* = 95 samples). Regurgitated pellets (castings) were also collected at the sample site but were not analyzed as part of this study. Only pluck sites and “larders” (areas near the nest where prey is stored; Walter, 1979) that could be associated unambiguously with nests were analyzed. Avian prey was primarily identified based on feathers using voucher specimens (personal collection of author YK) and relevant publications (Cieślak & Bolesław, 2006; Hansen et al., 1988; Hansen et al., 1991; Hansen et al., 1994; Hansen & Oelke, 1973; Hansen & Oelke, 1974; Hansen & Oelke, 1976; Hansen & Oelke, 1978; Hansen & Oelke, 1983). Wing remnants were used to identify insects (personal collection of author BS). This study was approved by the Ministère de l'Enseignement Supérieur et de la Recherche Scientifique (M.E.S.R.S.) and all procedures followed were in accordance with international ethical standards.

### 2.3 Autumn bird migration phenology

We grouped the birds preyed on by Eleonora's falcon from Kef Amor into four categories based on the timing of their capture: (1) early migrants; (2) mid‐season (end August–early October) migrants; (3) late‐season (end September–late October) migrants; and (4) migrant birds preyed upon across almost the entire breeding period (July–October).

### 2.4 Statistical analysis

The relationship between the median passage date of songbird migrants across the Curonian Spit of the Baltic Sea (Sokolov et al., 1999) and the capture date (sampling date) of avian prey items at Kef Amor in 2012 was determined by performing a linear regression analysis using the package {stats}. We added data from 2010 and 2011 for birds with a limited sample size. We also used a one‐way ANOVA test followed by a post‐hoc Tukey test to investigate significant differences in average species richness per nest across years. Analyses were conducted in the R software package, version 4.0.5 (R Development Core Team, 2021).

## 3 RESULTS

All 513 samples we assessed across the three breeding seasons contained feathers, but a few (*N* = 9 samples) also harbored insect remains. Overall, we identified 3080 individual avian prey items representing 77 unique avian prey species (Appendix S1: Table S1), mainly comprising songbirds (Passeriformes) (85.7%). The number of individual avian prey items varied between years: 1122 (2010), 656 (2011), and 1302 (2012). In addition, three species of insect (the death's head hawkmoth *Acherontia atropos* (*N* = 19 prey items), the dragonfly *Anax* sp. (*N* = 2 prey items), and the European rhinoceros beetle *Oryctes nasicornis* (*N* = 9 prey items)) and one reptile (the common wall gecko *Tarentola mauritanica*) were recorded as prey remains.

**FIGURE 2:**
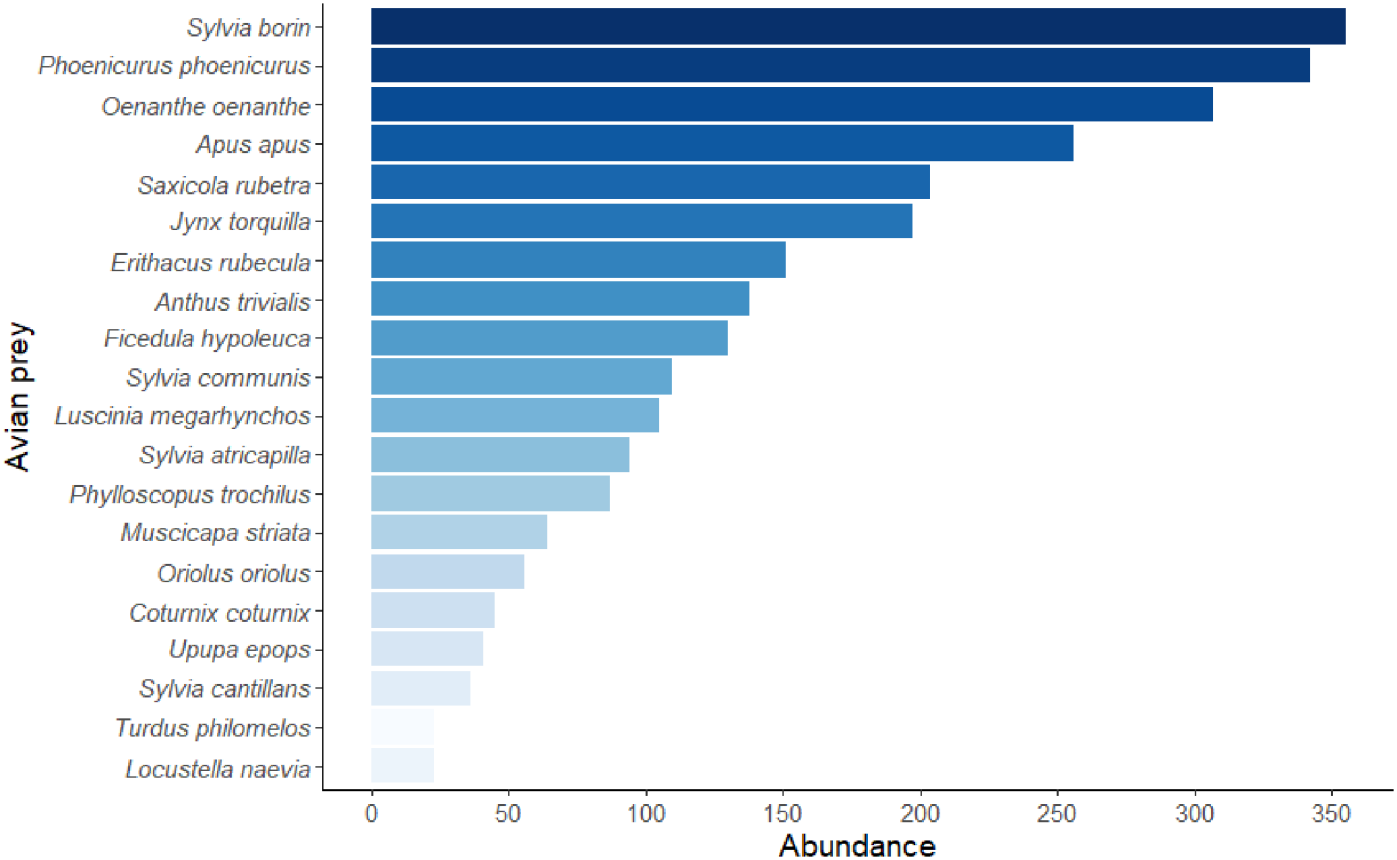
Abundances of the 20 most common bird migrants captured by Eleonora's falcon at Kef Amor during the study period (2010–2012)

The most abundant prey in decreasing order were the garden warbler *Sylvia borin*, the common redstart *Phoenicurus phoenicurus*, the northern wheatear *Oenanthe oenanthe*, the common swift *Apus apus*, and the whinchat *Saxicola rubetra* (Figure 2). Together, these five species accounted for almost half (47.5%) of all birds identified as prey of Eleonora's falcon. The 20 most numerous species accounted for 89.7% of all the identified avian prey.

**FIGURE 3:**
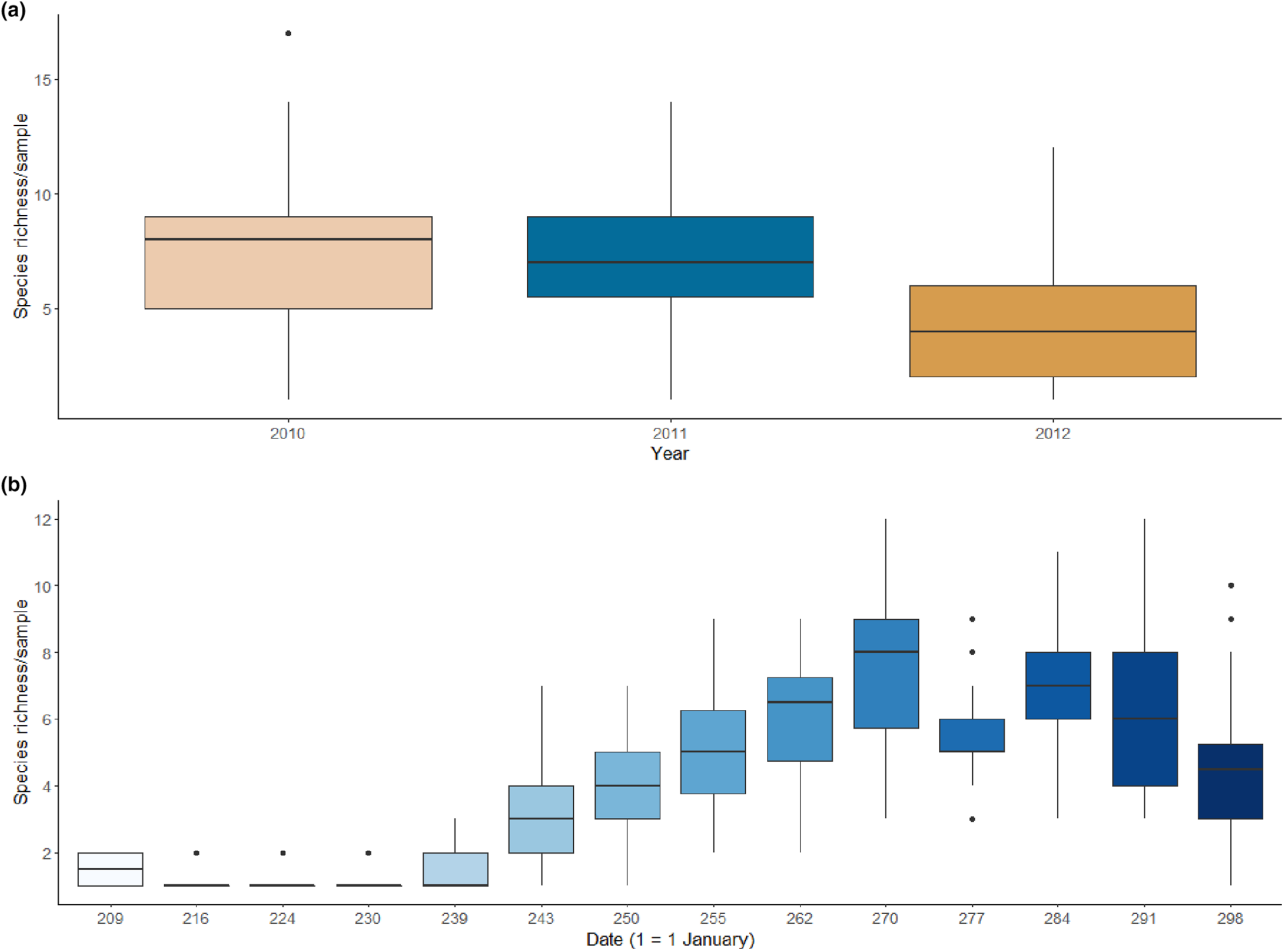
Annual variation in species richness of avian prey items per sample at Kef Amor (a). Weekly variation in species richness of avian prey items at Kef Amor from 20 July to 24 October 2012 (b). Sample size is given in parentheses

We observed a decrease in the diversity of migrant birds preyed upon by Eleonora's falcon between 2010 and 2012. The mean number of birds preyed upon per nest declined from 7.4 ± 3.2 (*N* = 134) in 2010 to 7.1 ± 2.6 (*N* = 91) in 2011 and even further to 4.3 ± 2.8 (*N* = 278) in 2012. Furthermore, the species richness of bird remains per nest also declined markedly in 2012 (Figure 3a). One‐way ANOVA and post‐hoc Tukey tests indicated significant differences in species richness between 2012 and the two preceding years (*F*_2,500_ = 67.11, *p* < .001). This decrease coincided with a marked reduction in overall number of bird prey species in 2012 (64 species in 2010 vs. 55 species in 2012). Migrant birds presenting the greatest decline as prey across years include the Eurasian golden oriole *Oriolus oriolus*, the subalpine warbler *Sylvia cantillans*, the hoopoe *Upupa epops*, the melodious warbler *Hippolais polyglotta*, the Eurasian reed warbler *Acrocephalus scirpaceus*, and the red‐backed shrike *Lanius collurio*.

**FIGURE 4:**
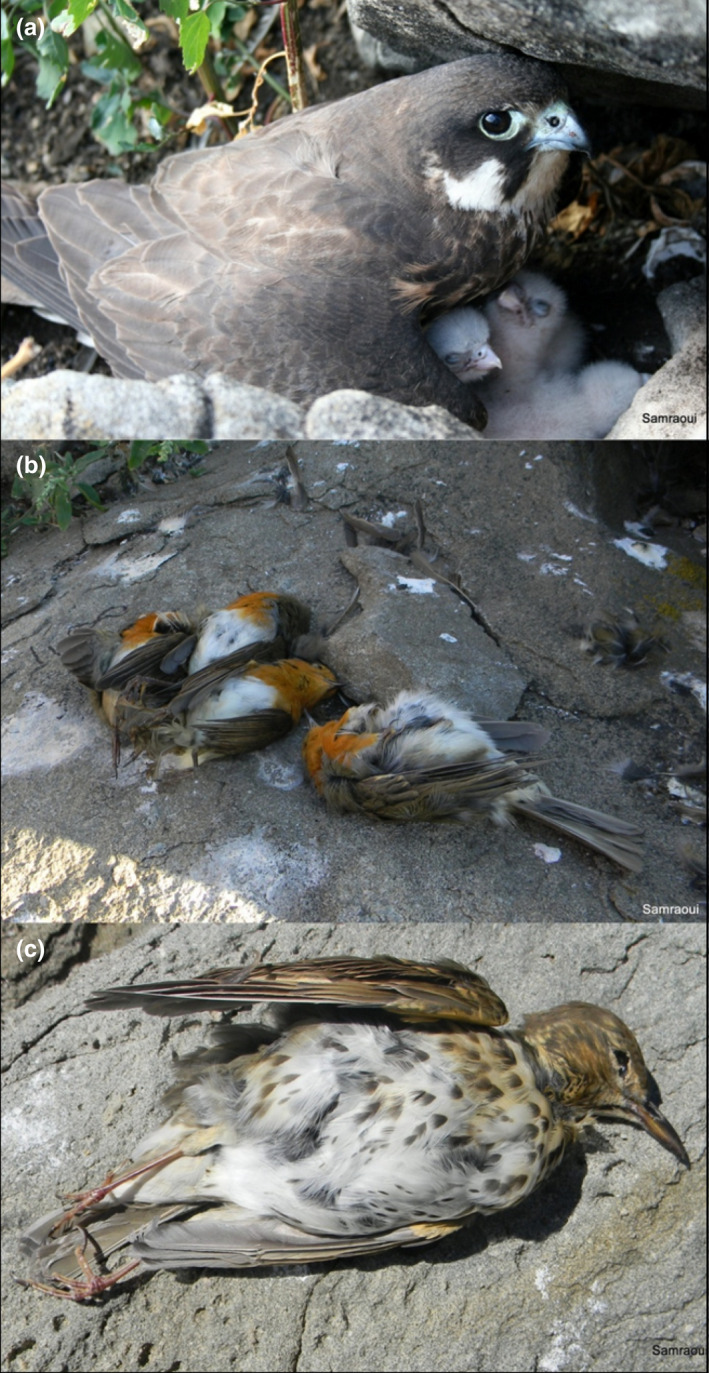
Eleonora's falcon with its nestlings (a), a “larder” stocked with European robins (b), and an intact song thrush kept for later consumption (c)

Moreover, we observed that species richness of prey items varied across the breeding season. During egg incubation (July to mid‐August), Eleonora's falcons in Northeast Algeria appear to rely principally on one or two prey species, primarily the common swift. Prey species richness gradually increases to a peak from the end of August (coinciding with the onset of hatching) to the end of September, before declining thereafter (Figure 3b). Prior to the fledging stage (October) (Figure 4a), the European robin *Erithacus rubecula* became a staple in the diet of Eleonora's falcons, with “larders” (Figure 4b,c) and nests frequently stocked with this prey species.

**FIGURE 5:**
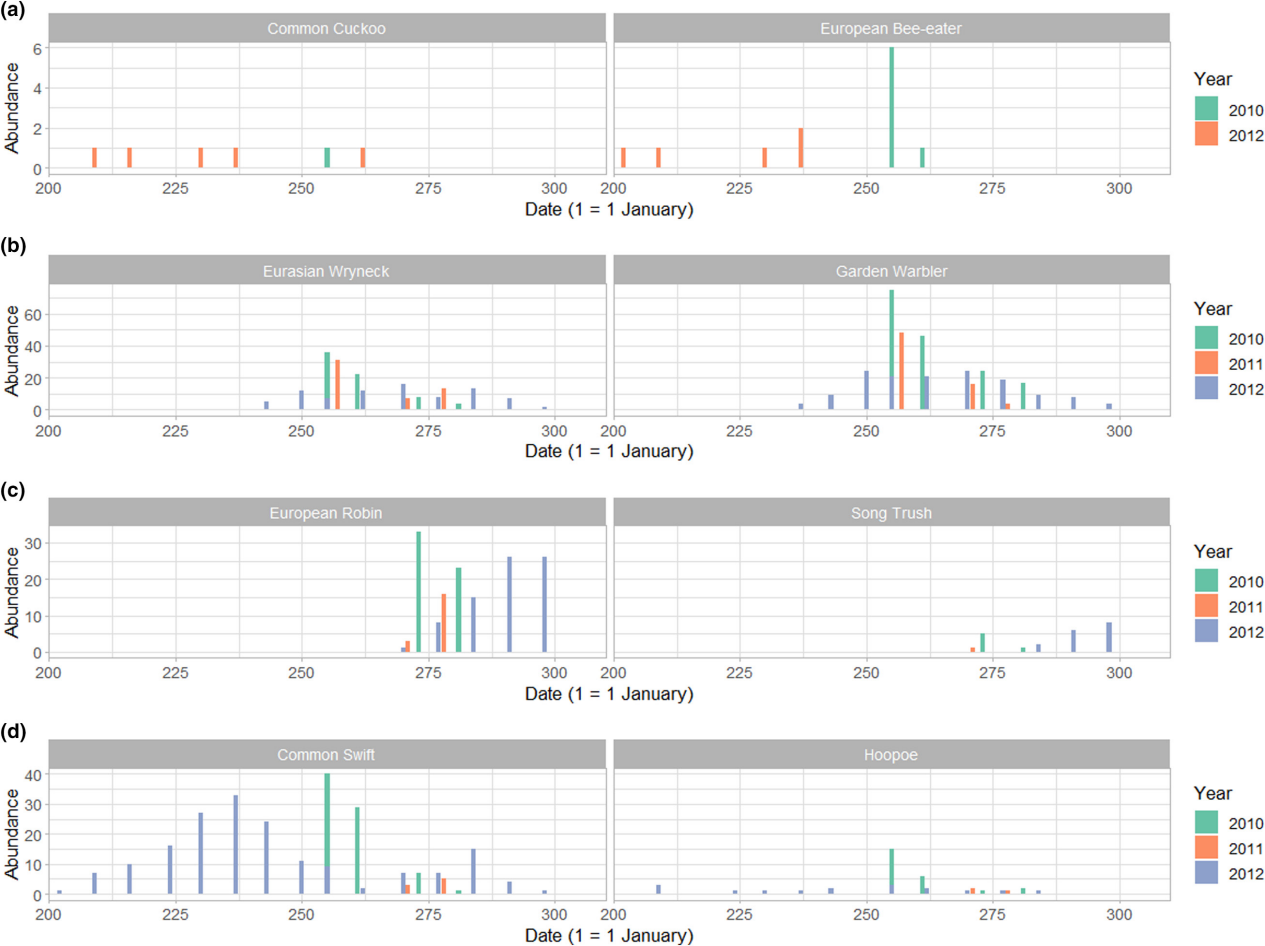
Distinct autumn migratory phenology exhibited by passerines preyed upon by Eleonora's falcon at Kef Amor. Two passerines were selected to represent each category: early (July and August) long‐distance migrants (a); mid‐season (end August–early October) long‐distance migrants (b); late‐season (end September–late October) medium‐ and short‐distance migrants (c); and migrant birds preyed upon across almost the entire breeding period (d)

Birds preyed on by Eleonora's falcon from Kef Amor could be classified into four categories based on their autumn migration phenology: (1) early long‐distance migrants such as the common cuckoo *Cuculus canorus* and the European bee‐eater *Merops apiaster* that are caught early in the nesting season (July and August) (Figure 5a); (2) mid‐season (end August–early October) long‐distance migrants such as Eurasian wryneck *Jynx torquilla* and garden warbler (Figure 5b); (3) late‐season (end September–late October) medium‐ and short‐distance migrants such as the European robin and song thrush *Turdus philomelos* (Figure 5c); and (4) migrant birds preyed upon across almost the entire breeding period (July–October), such as the common swift and the hoopoe (Figure 5d).

**FIGURE 6:**
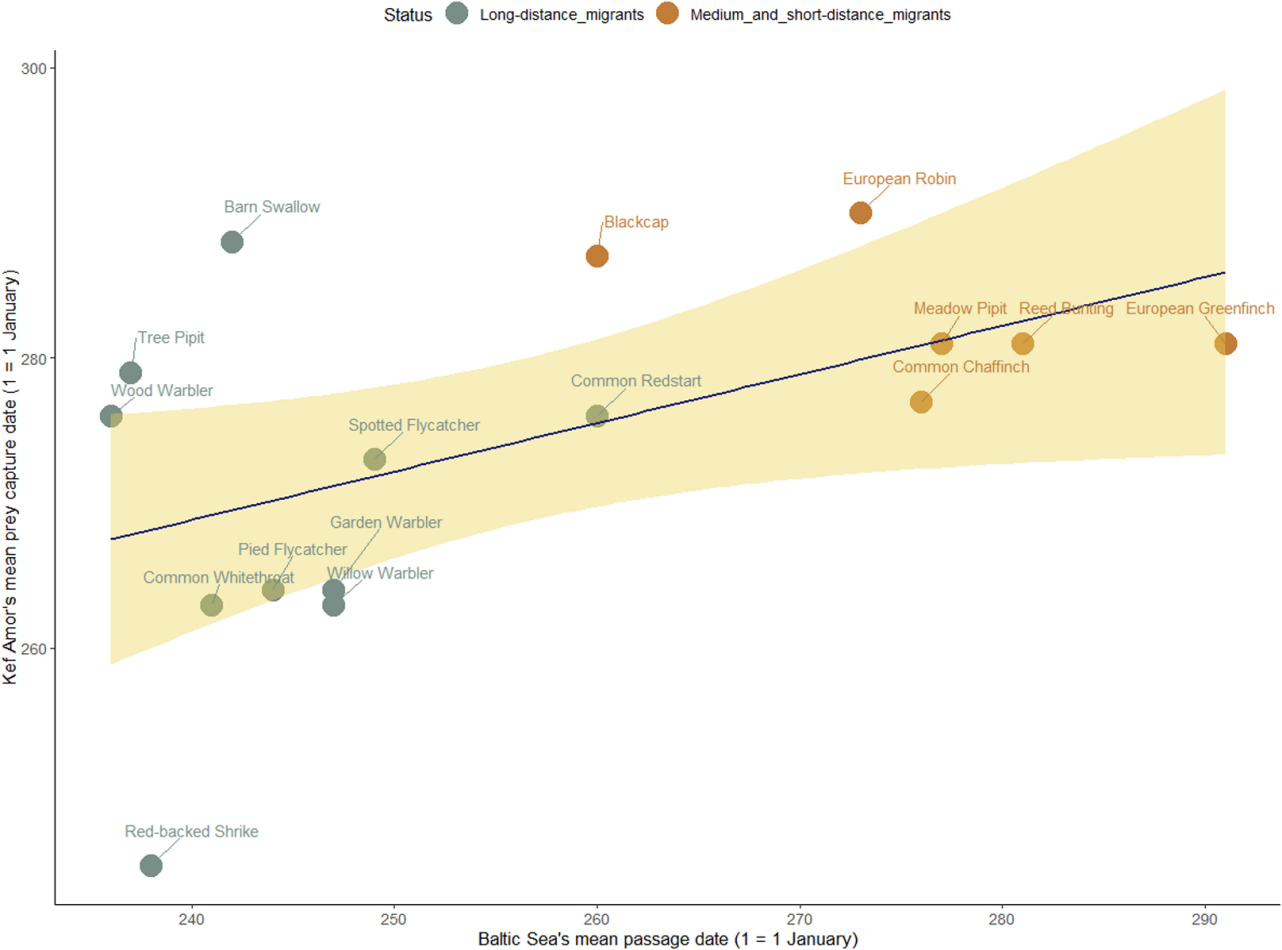
Relationship between mean passage data at the Baltic Sea (Sokolov et al., 1999) and mean prey capture date at Kef Amor in 2012 for 16 migratory songbirds. The blue line and shaded area represent a fitted linear regression and 95% confidence interval for the mean, respectively

A linear regression analysis of the mean date of capture for 16 passerine prey of Eleonora's falcon from Kef Amor in 2012 (dependent variable) and the mean date of passage of the same prey species recorded at the Curonian Spit of the Baltic Sea (as a predictor) (Samraoui & Samraoui, 2008) revealed a significant positive relationship suggesting that dietary studies of Eleonora's falcon may provide insights for the autumn avian migration phenology across a large spatial scale (Table 2; Figure 6). Figure 6 also highlighted the different timings for passage and capture between long‐distance and medium‐ to‐short‐distance migratory songbirds.

**TABLE 2:** Summary of linear regression model of Kef Amor sample dates for 16 passerines using Baltic Sea passage date (Sokolov et al., 1999) as a predictor variable

Parameter	Estimate	SE	*p*‐value
Intercept	193.69	28.03	7.22e−06
Baltic Sea dates	0.31	0.11	.01

## 4 DISCUSSION

Little is known about the diet of Eleonora's falcon in Algeria and the status of extant colonies (Bakour & Moulaï, 2019; Peyre et al., 2018; Touati et al., 2017). As reported in previous studies for other populations (Walter, 1979), we found that Eleonora's falcon is primarily an avian predator during the breeding season. An estimated 2.1 billion songbirds and near‐passerine birds use autumnal Palearctic–African migratory pathways each year (Hahn et al., 2009; Moreau, 1972), with one in every 2500 to 5000 migrants ending up as Eleonora's falcon prey as they cross the Mediterranean Sea (Walter, 1979). Although Eleonora's falcons may forage on the continental landmasses and prey on resident species there if islets where they nest are not far offshore (Ristow & Wink, 1995), our observations indicate that this behavior is uncommon during the breeding season at our study site in Algeria.

### 4.1 Study biases

This study is not exempt from limitations associated with previous research analyzing prey remains (Redpath et al., 2001; Robinson et al., 2018). Indirect collection of prey remains from raptors may be biased toward larger and brightly colored feathers. Importantly, predator dietary profiles may not always accurately reflect some aspects of the migratory patterns of their prey due to, for instance, an absence of nocturnal species, species that migrate in large protective groups, or prey preferences, and prey remains may not represent prey availability or migration densities since poor and good flyers may be over‐ or underrepresented, respectively (Walter, 1979). However, the results are encouraging, and given there has been little previous research regarding the relationship between dietary studies of the Eleonora's falcon and avian migrants' population dynamics, there is clearly a need to extend this study using pellets and stable isotopes. In addition, expanding the work to cover multiple sites over a long period would be most promising.

### 4.2 Prey composition

In addition to the clear dominance of songbirds in the diet of our study population of Eleonora's falcon, we identified a high number of prey species (*n* = 77) and marked temporal variation, providing evidence of strong dietary plasticity. Prey composition and abundance at Kef Amor closely match patterns reported for the same falcon species in Sardinia (Spina et al., 1987), being dominated by prey such as the garden warbler, common redstart, and northern wheatear, and to a lesser extent, common swift, whinchat, and Eurasian wryneck. However, our findings differ from those of previous studies conducted in western Algeria where pallid swifts *Apus pallidus* and subalpine warblers were dominant (Bakour & Moulaï, 2019), or in the Eastern Mediterranean where red‐backed shrikes, lesser‐gray Shrikes *Lanius minor*, common whitethroats *Sylvia communis*, and hoopoes were more common and where common redstarts were less prevalent (Walter, 1979; Xirouchakis et al., 2019).

The two most abundant prey items at Kef Amor were the garden warbler and the common redstart. The garden warbler winters in Equatorial Africa and occurs in Northern Algeria during its autumn migration (Bairlein, 1988, 1991; Isenmann & Moali, 2000). The common redstart is abundant in the Western Mediterranean Basin during its migration, with a peak in autumnal transit from mid‐September to mid‐October (Isenmann & Moali, 2000). Moreover, both the garden warbler and the common redstart are among the 10 most numerous long‐distance migrants on the Palearctic–African flyway (Hahn et al., 2009). Other relatively abundant migrants that fell prey to the Eleonora's falcon at Kef Amor were the willow warbler *Phylloscopus trochilus*, the tree pipit *Anthus trivialis*, the common whitethroat, and the pied flycatcher *Ficedula hypoleuca* (Hahn et al., 2009).

### 4.3 Miscellaneous prey

Our recovery of a small number of insect prey items seems to confirm a seasonal switch in the diet of this population of Eleonora's falcon, as reported previously for other raptors known to exhibit a seasonal dietary switch in accordance with prey availability (Breckenridge & Errington, 1938). We find it noteworthy that two of the three prey insects we identified are well known for their migratory habits. The death's head hawkmoth is a resident species in North Africa but also a regular summer migrant to Europe (Pittaway, 1993), and aeshnid dragonflies are powerful flyers. Dragonflies can represent a regular food item for Eleonora's falcon (Gallagher et al., 2017), but their availability may vary geographically (Bakour & Moulaï, 2019; Xirouchakis et al., 2019). For instance, lesser emperors *Anax parthenope* may cross the Mediterranean, and the Afrotropical migrant *Anax ephippiger* can even reach Britain and Iceland (Corbet, 1999). Our identification of wall gecko among Eleonora's falcon prey remains at Kef Amor contributes to the debate as to whether lizards constitute an element of their diet (Walter, 1979; Xirouchakis et al., 2019).

### 4.4 Variation in the diversity of migrant songbird prey

Given the heterogeneous nature of our sampling effort, we urge caution in interpreting the variation across years in abundance and species richness of songbird prey. Nevertheless, it is clear that overall species richness of bird remains declined between 2010 (for which we had far fewer samples) and 2012. Two widespread prey species that exhibited a marked decline between years at Kef Amor are the golden oriole and the red‐backed shrike, both of which may be on the verge of extinction as breeding species in parts of their range (Stanbury et al., 2017). It should be noted that dietary studies may be sensitive to natural population fluctuations over short spatial and timescales. Further investigations are warranted to ascertain if the recorded decline represents a stochastic event or a worrying trend. We are far more confident in asserting that there is seasonal variation in prey species richness, which reaches a peak in late September, as reported elsewhere (Xirouchakis et al., 2019).

### 4.5 Phenology

The phenology of the bird remains we identified at Kef Amor reflects the broad timings of passerine autumnal migration, as revealed by the seasonal change in songbird prey closely agreeing with analyses of ring recoveries from the Curonian Spit (Sokolov et al., 1999). Accordingly, we were able to classify prey into four main categories, i.e., early‐season migrants, mid‐season migrants, late‐season migrants, and birds that migrate throughout the falcon's breeding season. This classification, a reflection of the time constraint imposed by survival and reproduction on migrants, seems to be conserved over a broad scale (Winger & Pegan, 2021). The seasonal changes in prey composition we report herein confirm previous findings that Eleonora's falcon starts feeding on migrant birds well before their chicks hatch (Walter, 1979). At Kef Amor, we identified at least six species that fell prey in July 2012 (i.e., common swift, common cuckoo, European bee‐eater, hoopoe, and rufous‐tailed scrub robin *Cercotrichas galactotes*), which is 2 weeks before the first chick hatched. Similarly, at Mogador in Morocco, at least 10 bird species were found among prey caught by Eleonora's falcon in July, a full month before the start of the hatching period (Walter, 1979).

Two long‐distance migrants, the common cuckoo and European bee‐eater, were the earliest birds to fall prey to Eleonora's falcon at Kef Amor during the breeding season. Satellite tracking and ringing studies in Northern Europe indicate that emigrating first‐year common cuckoos peak in July–August, whereas adults tend to leave earlier (Seel, 1977; Vega et al., 2016). As an obligate brood parasite, the common cuckoo can depart early from its breeding ground to winter in tropical Africa since it does not have to devote attention to its chicks (Davies, 2015). In contrast, the early passage of the European bee‐eater we uncovered herein is surprising as it is known to begin southward migration in mid‐August, with a peak in September (Cramp, 1998). However, our findings do corroborate records from South Sinai, Egypt (Arcilla et al., 2016), and a study showing that distinct geographic populations of European bee‐eater may differ in their migratory phenology by 2–4 weeks (Hahn et al., 2020).

We identified two other trans‐Saharan migrants, the common swift and the Eurasian hoopoe, among samples obtained early in the breeding season (coinciding with the first hatchlings), i.e., in the second half of August. However, these species were also found throughout the rest of the breeding season, albeit their frequencies by mid‐September are superseded by those of other prey. Our data are congruent with knowledge of the chain migration of common swifts in Europe, whose departure from the breeding grounds (from early July to early September) is positively correlated with increasing breeding latitudes (Åkesson et al., 2020). We found that common swifts dominated other prey in the early stages of the breeding period of Eleonora's falcon at Kef Amor (July and August), as also reported for a population in Sardinia (Spina et al., 1987). Importantly, the migratory phenology of the common swift is strongly dependent on weather conditions, with departure dates potentially being delayed by adverse weather (Koskimies, 1961).

We found that the migratory time frame for some migrants (Eurasian wryneck, garden warbler, northern wheatear, common whitethroat, whinchat, and common nightingale *Luscinia megarhynchos*) was lengthy and coincided with the development of Eleonora's falcon chicks (hatching to fledging from end of August to October). For instance, the median autumnal trapping date for the garden warbler on Capri, Southern Italy, was 25 September (Ottosson et al., 2005). The protracted migration of the garden warbler is partly explained by its extensive stopover before crossing the Mediterranean Sea and age‐related differences in the timing of its autumn migration (Barboutis et al., 2014; Hall‐Karlsson & Fransson, 2008). Further north, passage of northern wheatears across the small Heligoland archipelago (Germany) ranges from late July to early November (Dierschke & Delingat, 2003). Thus, Eleonora's falcon chicks likely rely heavily on many migrant species such as garden warbler, northern wheatear, and whinchat to sustain their development.

In contrast, we found that Eleonora's falcon first preyed on European robins at the end of September, with that species becoming the principal prey item in the first weeks of October when falcon nestlings are about to fledge. That large numbers of European robins converge in North Africa during October is well documented (Erard, 1966; Remisiewicz, 2002), and this phenology is congruent with their known migration across the Baltic Sea (Högstedt & Persson, 1971; Saurola, 1983) and through southern Europe (Arizaga et al., 2010; Bottoni et al., 1991). The blackcap *Sylvia atricapilla* displays a similar pattern of migratory phenology, flying through Europe in September and October (Schubert et al., 1986). Owing to climate change and food subsidies (bird feeders), the migratory behaviors of European blackcap populations are changing, with novel migratory routes inducing rapid genetic and phenotypic divergences (Irwin, 2009; Rolshausen et al., 2009).

Another late migrant we identified as Eleonora's falcon prey was the song thrush. In Italy, migration of this species originating from the Baltic and Central‐Eastern Europe is highest between the end of October and early November (Andreotti et al., 1999; Busse & Maksalon, 1986). Other late‐migrating species (from late September/early October onwards) include the common starling, black redstart, greater short‐toed lark, and meadow pipit. Significantly, our data reveal the regular occurrence of late winter visitors of European greenfinch *Carduelis chloris* from Europe in Algeria, a phenomenon previously questioned (Isenmann & Moali, 2000).

Thus, the timing of prey capture by Eleonora's falcon supports previous findings indicating that long‐ and short‐distance migrants may adopt distinct migratory strategies (Bruderer & Salewski, 2009). Optimal migratory strategies involve a trade‐off between time, energy, and predation risks (Alerstam, 2011; Pomerov, 2006). However, the constraint of time selection during autumn migration for long‐distance migrants may be stronger prompting them to advance the timing of their departure (Nilsson et al., 2014). In the context of global change, understanding how this time constraint that may be the cause of the marked decline in long‐distance migrants is modulated by environmental changes is of paramount importance (Berthold & Fiedler, 2005; Vickery et al., 2014).

### 4.6 Conservation

A previous study highlighted the increasing anthropogenic pressures on Mediterranean colonies of Eleonora's falcon (Touati et al., 2017). However, disturbances at breeding sites may not be the only factor contributing to severe population declines, although investigations of factors exacerbating colony decline are often hampered by a lack of knowledge about prey species' wintering grounds and stopovers. Our results also underscore how the fate of Eleonora's falcon is intimately linked to that of its prey, which mainly comprises migrant songbirds during its nestling phase. Evidence is accumulating that migratory birds are responding to climate change (Walther et al., 2002), potentially contributing to population declines (Bairlein, 2016; Møller et al., 2008; Saino et al., 2011), which likely has negative knock‐on effects for Eleonora's falcon. Despite the limitation stemming from distinct populations intermingling at migration bottlenecks, dietary studies in the same way as capture data may have the potential to provide independent control of breeding bird surveys and productivity, inform on population declines, and contribute to bird conservation (Berthold, 2004; Maggini et al., 2021).

## AUTHOR CONTRIBUTIONS

**Yves Kayser:** Investigation (equal); writing – review and editing (equal). **Laïd Touati:** Investigation (equal); writing – review and editing (equal). **Farrah Samraoui:** Conceptualization (equal); project administration (equal); writing – review and editing (equal). **Abdennour Boucheker:** Investigation (equal); writing – review and editing (equal). **Hamed A. El‐Serehy:** Funding acquisition (equal); resources (equal); writing – review and editing (equal). **Kenz Raouf Samraoui:** Investigation (equal); software (equal); visualization (equal); writing – review and editing (equal). **Boudjéma Samraoui:** Conceptualization (equal); formal analysis (equal); methodology (equal); visualization (equal); writing – original draft (equal).

## CONFLICTS OF INTEREST

The authors declare no conflict of interest.

## Supporting information


Appendix S1

## Data Availability

Data are available upon request and are deposited in the Dryad repository: https://doi.org/10.5061/dryad.9w0vt4bj5
