# Supplementary material for: Diet of breeding Eleonora's falcon Falco eleonorae in Algeria: Insights for the autumn trans‐Mediterranean avian migration
Source: Ecol Evol. 2022 Jul 5;12(7):e9065. doi: 10.1002/ece3.9065 (PMC9254077; doi:10.1002/ece3.9065)
Supplement: Supplementary file 1 — Appendix S1 [file ECE3-12-e9065-s001.docx]

**SI Appendix**

**Table S1:** Checklist and abundances of recorded songbird prey species for Eleanora’s Falcon at Kef Amor during 2010-2012.

|  | Species | Code | 2010 | 2011 | 2012 | Total |
| --- | --- | --- | --- | --- | --- | --- |
| 1 | Garden Warbler *Sylvia borin* | Sbor | 144 | 68 | 143 | 355 |
| 2 | Common Redstart *Phoenicurus phoenicurus* | Ppho | 114 | 74 | 154 | 342 |
| 3 | Northern Wheatear *Oenanthe oenanthe* | Ooen | 117 | 75 | 115 | 307 |
| 4 | Common Swift *Apus apus* | Aapu | 74 | 8 | 174 | 256 |
| 5 | Whinchat *Saxicola rubetra* | Srub | 77 | 47 | 80 | 204 |
| 6 | Eurasian Wryneck *Jynx torquilla* | Jtor | 64 | 51 | 82 | 197 |
| 7 | European Robin *Erithacus rubecula* | Erub | 56 | 19 | 76 | 151 |
| 8 | Tree Pipit *Anthus trivialis* | Atri | 53 | 33 | 52 | 138 |
| 9 | Pied Flycatcher *Ficedula hypoleuca* | Fhyp | 33 | 39 | 58 | 130 |
| 10 | Common Whitethroat *Sylvia communis* | Scom | 32 | 38 | 40 | 110 |
| 11 | Common Nightingale *Luscinia megarhynchos* | Lmeg | 48 | 21 | 36 | 105 |
| 12 | Blackcap *Sylvia atricapilla* | Satr | 29 | 23 | 42 | 94 |
| 13 | Willow Warbler *Phylloscopus trochilus* | Ptro | 30 | 30 | 27 | 87 |
| 14 | Spotted Flycatcher *Muscicapa striata* | Mstr | 25 | 22 | 17 | 64 |
| 15 | Eurasian Golden Oriole *Oriolus oriolus* | Oori | 28 | 19 | 9 | 56 |
| 16 | Common Quail *Coturnix coturnix* | Ccot | 16 | 9 | 20 | 45 |
| 17 | Hoopoe *Upupa epops* | Uepo | 22 | 3 | 16 | 41 |
| 18 | Subalpine Warbler *Sylvia cantillans* | Scan | 18 | 9 | 9 | 36 |
| 19 | Common Grasshopper Warbler *Locustella naevia* | Lnae | 6 | 3 | 14 | 23 |
| 20 | Song Thrush *Turdus philomelos* | Tphi | 6 | 1 | 16 | 23 |
| 21 | Melodious Warbler *Hippolais polyglotta* | Hpol | 10 | 10 | 2 | 22 |
| 22 | Orphean Warbler *Sylvia hortensis* | Shor | 8 | 6 | 5 | 19 |
| 23 | Eurasian Reed Warbler *Acrocephalus scirpaceus* | Asci | 11 | 2 | 3 | 16 |
| 24 | Great Reed Warbler *Acrocephalus arundinaceus* | Aaru | 6 | 2 | 6 | 14 |
| 25 | Red-backed Shrike *Lanius collurio* | Lcol | 10 |  | 4 | 14 |
| 26 | European Bee-eater *Merops apiaster* | Mapi | 7 |  | 5 | 12 |
| 27 | Yellow Wagtail *Motacilla flava* | Mfla | 4 | 4 | 4 | 12 |
| 28 | Black-eared Wheatear *Oenanthe hispanica* | Ohis | 4 | 6 | 2 | 12 |
| 29 | Bluethroat *Luscinia svecica* | Lsve | 2 | 2 | 7 | 11 |
| 30 | Spotted Crake *Porzana porzana* | Ppor | 1 | 2 | 8 | 11 |
| 31 | Sardinian Warbler *Sylvia melanocephala* | Smel | 5 | 6 |  | 11 |
| 32 | Greater Short-toed Lark *Calandrella brachydactyla* | Cbra |  |  | 10 | 10 |
| 33 | Willow Warbler/Chiffchaff *Phylloscopus trochilus/collybita* | Pcol | 8 | 1 |  | 9 |
| 34 | Common Starling *Sturnus vulgaris* | Svul | 1 | 1 | 7 | 9 |
| 35 | Sky Lark *Alauda arvensis* | Aarv | 1 | 1 | 6 | 8 |
| 36 | Egyptian Nightjar *Caprimulgus europaeus* | Ceur | 3 |  | 5 | 8 |
| 37 | Western Bonelli's Warbler *Phylloscopus bonelli* | Pbon | 1 | 4 | 3 | 8 |
| 38 | Woodchat Shrike *Lanius senator* | Lsen | 1 |  | 6 | 7 |
| 39 | Warbler *Phylloscopus sp.* | Psp. |  |  | 7 | 7 |
| 40 | Pallid Swift *Apus pallidus* | Apal | 3 |  | 3 | 6 |
| 41 | Common Cuckoo *Cuculus canorus* | Cuca | 1 |  | 5 | 6 |
| 42 | Ortolan Bunting *Emberiza hortuluna* | Ehor | 4 | 1 | 1 | 6 |
| 43 | Savi's Warbler *Locustella luscinioides* | Llus | 1 | 5 |  | 6 |
| 44 | European Turtle Dove *Streptopelia turtur* | Stur | 3 |  | 3 | 6 |
| 45 | Tawny Pipit *Anthus campestris* | Acam | 5 |  |  | 5 |
| 46 | Black Redstart *Phoenicurus ochruros* | Poch | 4 |  | 1 | 5 |
| 47 | Rufous-tailed Rock Thrush *Monticola saxatilis* | Msax | 1 | 3 |  | 4 |
| 48 | Common Stonechat *Saxicola torquata* | Stor |  | 2 | 2 | 4 |
| 49 | Sedge Warbler *Acrocephalus schoenobaenus* | Asch |  | 2 | 1 | 3 |
| 50 | Corn Bunting *Emberiza calandra* | Ecal | 2 |  | 1 | 3 |
| 51 | Cirl Bunting *Emberiza cirlus* | Ecir | 3 |  |  | 3 |
| 52 | Common Chaffinch *Fringilla coelebs* | Fcoe | 1 |  | 2 | 3 |
| 53 | Barn Swallow *Hirundo rustica* | Hrus |  | 1 | 2 | 3 |
| 54 | Sandwich Tern  *Sterna sandvicensis* | Ssan |  | 2 |  | 2 |
| 55 | Meadow Pipit *Anthus pratensis* | Apra | 2 |  |  | 2 |
| 56 | Alpine Swift *Apus melba* | Amel | 2 |  |  | 2 |
| 57 | Rufous-tailed Scrub Robin *Cercotrichas galactotes* | Cgal |  |  | 2 | 2 |
| 58 | House Martin *Delichon urbica* | Durb | 1 |  | 1 | 2 |
| 59 | Eurasian Scops Owl *Otus scops* | Osco |  |  | 2 | 2 |
| 60 | Wood Warbler *Phylloscopus sibilatrix* | Psib | 1 | 1 |  | 2 |
| 61 | Water Rail *Rallus aquaticus* | Raqu | 2 |  |  | 2 |
| 62 | Common Blackbird *Turdus merula* | Tmer |  |  | 2 | 2 |
| 63 | Lesser Short-toed Lark *Calandrella rufescens* | Cruf |  |  | 1 | 1 |
| 64 | Dunlin *Calidris alpina* | Calp | 1 |  |  | 1 |
| 65 | Red Knot *Calidris canutus* | Ccnu | 1 |  |  | 1 |
| 66 | Little Stint *Calidris minuta* | Cmin | 1 |  |  | 1 |
| 67 | Common Linnet *Carduelis cannabina* | Ccan |  |  | 1 | 1 |
| 68 | European Greenfinch *Carduelis chloris* | Cchl | 1 |  |  | 1 |
| 69 | Black Tern *Chlidonias niger* | Cnig | 1 |  |  | 1 |
| 70 | Hawfinch *Coccothraustes coccothraustes* | Ccoc | 1 |  |  | 1 |
| 71 | Reed Bunting *Emberiza schoeniclus* | Esch | 1 |  |  | 1 |
| 72 | Western Olivaceous Warbler *Hippolais opaca* | Hopa | 1 |  |  | 1 |
| 73 | Red-rumped Swallow *Hirundo daurica* | Hdau | 1 |  |  | 1 |
| 74 | Little Crake *Porzana parva* | Ppar |  |  | 1 | 1 |
| 75 | Goldcrest/Firecrest *Regulus sp.* | Regu |  |  | 1 | 1 |
| 76 | Sand Martin *Riparia riparia* | Rrip | 1 |  |  | 1 |
| 77 | Redwing *Turdus iliacus* | Tili | 1 |  |  | 1 |
